# Comorbidity and Sex-Related Differences in Mortality in Oxygen-Dependent Chronic Obstructive Pulmonary Disease

**DOI:** 10.1371/journal.pone.0035806

**Published:** 2012-04-26

**Authors:** Magnus P. Ekström, Claes Jogréus, Kerstin E. Ström

**Affiliations:** 1 Department of Respiratory Medicine & Allergology, Institution for Clinical Sciences, University of Lund, Lund, Sweden; 2 Department of Mathematics and Science, School of Engineering, Blekinge Institute of Technology, Karlskrona, Sweden; 3 Department of Respiratory Medicine & Allergology, Institution for Clinical Sciences, University of Lund, Lund, Sweden; Leiden University Medical Center, The Netherlands

## Abstract

**Background:**

It is not known why survival differs between men and women in oxygen-dependent chronic obstructive pulmonary disease (COPD). The present study evaluates differences in comorbidity between men and women, and tests the hypothesis that comorbidity contributes to sex-related differences in mortality in oxygen-dependent COPD.

**Methods:**

National prospective study of patients aged 50 years or older, starting long-term oxygen therapy (LTOT) for COPD in Sweden between 1992 and 2008. Comorbidities were obtained from the Swedish Hospital Discharge Register. Sex-related differences in comorbidity were estimated using logistic regression, adjusting for age, smoking status and year of inclusion. The effect of comorbidity on overall mortality and the interaction between comorbidity and sex were evaluated using Cox regression, adjusting for age, sex, Pa_O2_ breathing air, FEV_1_, smoking history and year of inclusion.

**Results:**

In total, 8,712 patients (55% women) were included and 6,729 patients died during the study period. No patient was lost to follow-up. Compared with women, men had significantly more arrhythmia, cancer, ischemic heart disease and renal failure, and less hypertension, mental disorders, osteoporosis and rheumatoid arthritis (P<0.05 for all odds ratios). Comorbidity was an independent predictor of mortality, and the effect was similar for the sexes. Women had lower mortality, which remained unchanged even after adjusting for comorbidity; hazard ratio 0.73 (95% confidence interval, 0.68–0.77; P<0.001).

**Conclusions:**

Comorbidity is different in men and women, but does not explain the sex-related difference in mortality in oxygen-dependent COPD.

## Introduction

Severe Chronic Obstructive Pulmonary Disease (COPD) can be a devastating illness with high morbidity and mortality. [1] Long-term oxygen therapy (LTOT) is an established treatment in hypoxic (oxygen-dependent) COPD, as it was shown to approximately double survival in two randomized trials. [2], [3] The incidence of LTOT has increased markedly in women; in fact, the majority of patients starting LTOT in Sweden are now women. [4] Studies of sex-related differences in survival on LTOT for COPD have shown conflicting results. Women had better survival than men after starting LTOT in most studies, [4], [5], [6], [7] but in a study of Machado et al., survival was worse in women than men. [8] These inconsistent findings could be related to the heterogeneity of research settings, patient selection and covariates included in the analyses. Co-existing diseases, or comorbidity, was only evaluated in two of the studies, which both found a high overall prevalence of comorbidity but did not report any significant differences in comorbidity between men and women. [7], [8] Interestingly, one study found that comorbidity predicted mortality, but only in women. [7] It remains unknown whether the effect of comorbidity on mortality differs between men and women, as this has only been evaluated in one previous, relatively small, study, which found no effect measure interaction between comorbidity and sex. [8]

The prevalence of comorbidity in COPD is high, [9] and increases with age and COPD severity. [10], [11] Comorbidity has been shown to predict mortality in COPD, [11], [12] and may be more prevalent in men than in women. [11], [13] An analysis from the TORCH study, in which patients with significant comorbidity were excluded, found no significant difference in mortality between men and women. [14] In a study of patients matched in terms of COPD severity, [13] mortality was found to be higher in men than in women, and comorbidity to predict mortality in men only. This study formulated the hypothesis that the higher mortality in men was attributable to sex-dependent differences in comorbidity, especially in severe COPD. [14]

The present national prospective study evaluates whether there are differences in comorbidity between men and women in oxygen-dependent COPD, and tests the hypothesis that comorbidity contributes to sex-related differences in mortality.

## Materials and Methods

Patients aged 50 years or older, starting LTOT for physician diagnosed COPD between 1 January 1992 and 31 December 2008 in the Swedish National Oxygen Register were included prospectively. The indications for LTOT and details of the register have been reported previously, [4] and part of the database was used in recently published studies. [15], [16] For patients who had started LTOT more than once (n = 99), only the latest treatment episode was included in the analysis. At baseline, defined as the date of starting LTOT, data were collected on arterial gas tension of oxygen (Pa_O2_) and carbon dioxide (Pa_CO2_) when breathing air and during oxygen therapy, forced expiratory volume in one second (FEV_1_), forced vital capacity (FVC), concomitant home mechanical ventilator use, smoking history (never, past or current smoking), the prescribed oxygen dose, and the prescribed duration of oxygen therapy per day.

Discharge diagnoses, the number of hospitalizations and in-hospital days within five years prior to starting LTOT were collected for all patients from the Swedish Hospital Discharge Register, which covers more than 98% of all hospitalizations in Sweden after 1 January 1987. [17] In addition, data on all lung transplantations and lung volume reduction procedures before starting LTOT and during follow up was collected.

Patients were followed until withdrawal of LTOT, death or 31 December 2008, whichever came first. Vital status and underlying cause of death was obtained from the Swedish Causes of Death Register. All diagnoses, procedure codes, and causes of death were coded according to the ninth (before 1997) and tenth revisions of the International Classification of Disease (ICD). [18], [19] Definitions of all diagnosis entities and procedures are found in a supporting information table (Table S1).

### Ethics statement

All patients gave their informed written consent to participate. The study was approved by the Lund University Research Ethics Committee (157/2007 and 350/2008), the Swedish National Board of Health and Welfare, and the Data Inspection Board.

### Statistical methods

Differences in baseline data were tested using chi square-test, Student's t-test, and Wilcoxon's rank-sum test for categorical, normally and non-normally distributed variables. Comorbidities were defined as relevant registered discharge diagnoses, primary or secondary, within five years prior to starting LTOT. In the analyses, comorbidity was categorized into the Charlson comorbidity score, [20] with COPD removed from the list of weighted diagnoses, in order to only include comorbidities of COPD. Age was not incorporated in the Charlson score and was included as an independent variable in all analyses.

Odds ratios for women as compared to men for individual comorbidities and for the Charlson score, adjusted for age, smoking history and start year, were analyzed using logistic regression and a generalized ordered logit model. [21] The ordered odds ratio for Charlson score was interpreted as the odds ratio of having a higher score than the present category. [21] The Hosmer-Lemeshow test was used to test the fit of the logistic models. [22]

The effect of comorbidity, categorized as Charlson score and individual diagnoses not included in the score, on overall mortality was evaluated using Cox regression, [23] adjusted for age, sex, Pa_O2_ breathing air, FEV_1_ (included as absolute values or percent of predicted, [24] respectively), start year and smoking history. The effect of adding body mass index (BMI) to the model was evaluated in the cohort starting LTOT 1995–2008, as BMI was registered during this time period only. Interactions between comorbidity and sex, age and start year, respectively, and between age and sex were analyzed on the multiplicative scale by entering interaction terms into the fully adjusted model. Biological interaction between sex and comorbidity (defined as a Charlson score of one or higher) on the additive scale was explored by calculating the adjusted attributable proportion, which is the proportion of the relative risk among exposed that is attributable to the interaction, [25] adjusted for all covariates in the Cox regression. The attributable risk among all men was calculated as: [the attributable proportion]×[the prevalence of Charlson score one or more among men].

The proportional hazards assumption of the Cox model was assessed graphically with log-log plots and by testing for interaction with analysis time for all covariates. The overall fit of the model was assured using Cox-Snell residuals.

All statistical tests were two-sided and P-values below the standard value of 0.05 were considered statistically significant. All confidence intervals were 95% intervals. All statistical analyses were performed with Stata version 11.1 (StataCorp LP; College Station, TX).

## Results

A total of 8,712 patients, 3,929 men and 4,783 (54.9%) women were included in the study after exclusion of 15 patients (0.2%) because of data irregularities. Concomitant home mechanical ventilator was used at baseline by 2% of both women and men. Lung transplantation was performed in only 7 patients before starting LTOT, and in 45 (0.5%) patients, 29 women and 16 men, during follow up. Only 60 patients (27 women) had had lung volume reduction surgery before starting LTOT, and during follow up, it was performed in 17 patients (8 women). The cohort was followed for a total of 20718.7 person-years with a median follow-up of 1.65 years (range 0–16.85 years). LTOT was withdrawn before death in 415 patients (4.8%), mainly owing to improved oxygenation. No patient was lost to follow up. In total, 6,729 patients, 3,199 men and 3,530 women, died under observation. Nonrespiratory causes of deaths were slightly more common in men than women (31.1% vs 27.4%; P = 0.001). Baseline characteristics are shown in Table 1. Compared with men, women were younger, had a lower mean absolute FEV_1_ but similar FEV_1_% of predicted, higher P_aCO2_, and more in-hospital days, although the median number of hospitalizations were the same as for men. In the subset of patients starting LTOT 1995 or later (n = 7593), the mean BMI [± SD] was 23.0±5.1 and 23.5±6.7 kg/m^2^ in men and women, respectively.

**Table 1:** Baseline characteristics of patients starting long-term oxygen therapy for chronic obstrucitve pulmonary disease, 1992–2008.

Characteristic	Women, n = 4,783 (55%)	Men, n = 3,929	P-value
Age, years	71.8±8.4	73.7±7.8	<0.001
Pa_O2_ air, kPa	6.5±0.9	6.7±0.9	<0.001
Pa_CO2_ air, kPa	6.5±1.2	6.1±1.2	<0.001
Pa_O2_ oxygen, kPa	8.8±1.1	8.9±1.1	<0.001
Pa_CO2_ oxygen, kPa	6.8±1.2	6.3±1.2	<0.001
FEV_1_, L	0.7±0.3	1.0±0.5	<0.001
FEV_1_ of predicted, %	34.0±17.0	35.5±19.6	0.005
FVC, L	1.5±0.5	2.2±0.9	<0.001
Smoking history, %			
Never	6	4	<0.001
Past	91	94	<0.001
Current	3	2	0.129
All Hospitalizations, n*	5 (3–9)	5 (3–9)	0.013
Hospitalizations for COPD, n*	2 (1–4)	2 (1–4)	<0.001
In-hospital days*	48 (25–87)	41 (21–77)	<0.001

Data presented as mean ± SD unless otherwise specified.

*Definition of abbreviations:* FEV_1_ = forced expiratory volume in one second; FVC = forced vital capacity; Pa_CO2_ air = arterial blood gas tension of carbon dioxide while breathing ambient air; Pa_CO2_ oxygen = arterial blood gas tension of carbon dioxide while breathing oxygen; Pa_O2_ air = arterial blood gas tension of oxygen while breathing ambient air; Pa_O2_ oxygen = arterial blood gas tension of oxygen while breathing oxygen.

* Hospitalizations and in-hospital days within 5 years prior to starting LTOT, presented as median (first quartile – third quartile).

Of all patients, 98.3% had been hospitalized at least once within five years prior to starting LTOT and 75% three or more times. The patients who had no hospitalization (1.7%) were evenly distributed between men (N = 77) and women (n = 73).

### Sex-related differences in comorbidity

As shown in Table 2, women had more hypertension, mental disorders, osteoporosis and rheumatoid arthritis, but less arrhythmias, cancer, ischemic heart disease and renal failure, as compared to men. There were no significant sex-related differences for anemia, cerebrovascular disease, diabetes mellitus, digestive organ disease, heart failure, lung cancer or pulmonary embolism. Men were more likely to have high Charlson scores than women, and this sex-related difference increased with each additional category in the Charlson score (Table 3).

**Table 2:** Prevalence and sex-related odds of comorbidity in patients starting long-term oxygen therapy for chronic obstructive pulmonary disease, 1992–2008.

Diagnosis	Prevalence in Women, n (%)	Prevalence in Men, n (%)	Adjusted Odds Ratio for Women Compared to Men*	95% Confidence Interval	P-value
All cancers†	460 (10)	503 (13)	0.74	0.64–0.84	<0.001
Lung cancer	76 (2)	79 (2)	0.74	0.53–1.02	0.065
Anaemia	245 (5)	183 (5)	1.11	0.90–1.36	0.329
Arrhythmia†	578 (12)	662 (17)	0.72	0.63–0.81	<0.001
Cerebrovascular disease	236 (5)	214 (5)	0.88	0.73–1.08	0.224
Diabetes mellitus	414 (9)	369 (9)	0.87	0.74–1.01	0.062
Digestive organ disease	922 (19)	831 (21)	0.90	0.81–1.01	0.062
Heart failure	1543 (32)	1254 (32)	1.09	0.99–1.20	0.072
Hypertension†	673 (14)	429 (11)	1.26	1.10–1.1.45	0.001
Ischemic heart disease†	864 (18)	880 (22)	0.77	0.69–0.86	<0.001
Mental disorder†	482 (10)	291 (7)	1.27	1.09–1.49	0.003
Osteoporosis†	485 (10)	131 (3)	3.42	2.80–4.19	<0.001
Pulmonary embolism	114 (2)	89 (2)	0.99	0.74–1.32	0.931
Renal failure†	72 (2)	101 (3)	0.58	0.42–0.79	0.001
Rheumatoid arthritis†	103 (2)	52 (1)	1.56	1.10–2.20	0.012

Percentages may not add up to 100 due to rounding.

* Odds ratio adjusted for age, smoking history and year of starting long-term oxygen therapy.

† Entities with significant sex-related difference in odds.

**Table 3:** Prevalence and sex-related odds of Charlson score in patients starting long-term oxygen therapy for chronic obstructive pulmonary disease, 1992–2008.

Charlson score	Prevalence in Women, n (%)	Prevalence in Men, n (%)	Adjusted Odds Ratio for Women Compared to Men*	95% Confidence Interval	P-value
0	2440 (51)	1962 (50)	1.00	0.91–1.09	0.921
1	1371 (29)	1012 (26)	0.79	0.71–0.88	<0.001
2	616 (13)	517 (13)	0.62	0.53–0.72	<0.001
3	224 (5)	235 (6)	0.49	0.39–0.62	<0.001
>3	132 (3)	203 (5)	-	-	-

Percentages may not add up to 100 due to rounding.

* Generalized ordered odds ratio adjusted for age, smoking history and year of inclusion. It is interpreted as the odds ratio of having a higher score than the present category.

### Comorbidity and mortality

Comorbidity was an independent predictor of adjusted mortality, with increasing mortality for each additional category in the Charlson score (Table 4). In addition, anemia, arrhythmia, mental disorders and osteoporosis, which are not included in the Charlson score, independently predicted mortality. When individual diagnoses were entered in the model instead of the Charlson score, the comorbidities which were statistically significant and increased mortality with 10% or more were anemia, arrhythmia, cancer, ischemic heart disease, heart failure, mental disorder, and osteoporosis (Table 5).

**Table 4:** Cox regression of overall mortality in patients starting long-term oxygen therapy, 1992–2008.

Characteristic	Hazard ratio	95% confidence interval	P-value
Age	1.04	1.04–1.04	<0.001
Female	0.73	0.68–0.77	<0.001
Pa_O2_ air	0.88	0.86–0.91	<0.001
FEV_1_	0.82	0.76–0.88	<0.001
Past smoking*	1.05	0.94–1.20	0.356
Current smoking*	1.33	1.07–1.64	0.010
Start year	1.00	1.00–1.01	0.391
Charlson score†			
1	1.14	1.07–1.22	<0.001
2	1.39	1.26–1.52	<0.001
3	1.52	1.32–1.75	<0.001
>3	1.69	1.42–2.02	<0.001
Anemia	1.28	1.10–1.48	0.001
Arrhythmia	1.23	1.13–1.34	<0.001
Mental disorder	1.18	1.06–1.32	0.003
Osteoporosis	1.31	1.17–1.48	<0.001

*Definition of abbreviations:* FEV_1_ = forced expiratory volume in one second; Pa_O2_ air = arterial blood gas tension of oxygen while breathing ambient air.

* Never smoking is used as reference category.

† Charlson score 0 is used as reference category.

**Table 5:** Cox regression of the effects of individual comorbidities on overall mortality.

Characteristic	Hazard ratio	95% confidence interval	P-value
Age	1.04	1.04–1.04	<0.001
Female	0.72	0.68–0.77	<0.001
P_aO2_ air	0.88	0.85–0.91	<0.001
FEV_1_	0.81	0.75–0.87	<0.001
Past smoking*	1.08	0.95–1.23	0.228
Current smoking*	1.36	1.10–1.69	0.005
Start year	1.00	1.00–1.01	0.300
All cancers	1.28	1.17–1.41	<0.001
Anemia	1.31	1.12–1.52	<0.001
Arrhythmia	1.24	1.14–1.36	<0.001
Cerebrovascular disease	1.01	0.88–1.16	0.914
Diabetes mellitus	1.11	1.00–1.24	0.060
Heart failure	1.10	1.03–1.17	0.006
Hypertension	1.03	0.93–1.14	0.591
Ischemic heart disease	1.12	1.04–1.21	0.004
Mental disorder	1.17	1.05–1.31	0.005
Osteoporosis	1.30	1.15–1.47	<0.001
Pulmonary embolism	1.20	0.96–1.50	0.101
Renal failure	1.11	0.87–1.43	0.394
Rheumatoid arthritis	1.15	0.93–1.42	0.197

* Never smoking is used as reference category.

The crude overall mortality was lower for women than for men, hazard ratio (HR) 0.76 (95% confidence interval (CI), 0.73–0.80; P<0.001). As shown in Table 4 and 5, this survival benefit for women was not explained by comorbidity, since it remained relatively unchanged in multiple regression models which adjusted for Charlson score and individual comorbidities, as well as the other covariates. The sex-related difference remained unchanged even after adjusting, in addition to the covariates above, for the number of hospitalizations in the preceding five years or when excluding patients with no prior hospitalization, HR 0.73 (95% CI, 0.68–0.77; P<0.001) and HR 0,73 (95% CI, 0.68–0.78; P<0.001), respectively. In addition, the model estimates remained unchanged when including BMI and FEV_1_ in percent of predicted instead of the absolute values, as shown in the supplemental Table S2. There were no signs of multiplicative interaction between comorbidity and sex; P-values for interaction terms ranging 0.669–0.842, or comorbidity and age (P ranging 0.108–0.924), comorbidity and start year (P ranging 0.100–0.862), or age and sex (P ranging, 0.436–0.892). Although not statistically significant, data indicated a small biological interaction between comorbidity and sex on the additive scale, attributable proportion 0.073 (95% CI; −0.01, 0.16). Thus, interaction may have attributed to 7.3% of the relative risk in men with comorbidity, and 3.7% of the relative risk among all men.

## Discussion

The main findings in the present study are that in oxygen-dependent COPD 1) comorbidity differs between men and women, 2) comorbidity is an independent predictor of mortality with similar effects for both sexes, and 3) comorbidity does not explain the survival difference between men and women.

To the authors' knowledge, this is the first study to evaluate sex-related differences in the prevalence of individual diagnosis entities in a large cohort of patients with oxygen-dependent COPD. The only two previous studies of sex-stratified comorbidity in the field included relatively few patients and found no significant difference in the mean number of comorbidities [7] or Charlson score, [8] respectively.

The higher prevalence of cardiovascular disease among men is consistent with previous studies of patients with a broader range of COPD-severity. [11], [26] The finding of more hypertension among women than men is consistent with some previous studies, [26], [27] but not all. [11] The lack of sex-related difference in heart failure in the present study, in spite of a previous report of a higher prevalence among men, [11] might be due to the differences in patient selection and the particular difficulty of detecting heart failure in severe COPD. For this reason, the heart failure estimates should be interpreted with caution. Interestingly, mental disorders were more common among women than men, supporting recent findings that female sex is a determinant of depression in COPD. [28]

In the present study, comorbidity predicted mortality, independent of age, sex, degree of hypoxia, FEV_1_, year of starting LTOT, and smoking status. Previous studies of whether Charlson score predicts mortality in COPD have shown conflicting results, [7], [8], [10], [11], [12], [13], [29], [30], [31] which may be due to relatively small study populations, [7], [29] inclusion of few women, [12], [29], [30] or inclusion of covariates that captured part of the effect of comorbidity in the multiple regression models, such as dyspnea, measures of physical capacity and quality of life, [8], [13] which may have biased the estimates for comorbidity toward less effect. Although often used, the Charlson score has not been validated in COPD. In the present study, several comorbidities predicted mortality independent of the Charlson score, which was also found in another recent study. [31] This highlights the need for an updated comorbidity score validated specifically for COPD.

A previous study of our group showed that women had a better adjusted survival than men in oxygen-dependent COPD, but that this unexplained survival advantage for women also exists in the Swedish general population. [16] In fact, women had higher relative mortality than men, when the mortality was compared with the expected mortality in the general population for women and men respectively. [16] The driving hypothesis behind the present study was that differences in comorbidity, which was not evaluated in the previous study, explain part of the survival advantage for women compared with men after starting LTOT for COPD. [4], [5], [6], [7] Surprisingly, the present findings strongly argue against any such effect on the sex-related difference in survival. Furthermore, the effect of comorbidity on the mortality rate-ratio was not modified by sex, and any biological interaction was small. Thus, the longer survival for women as compared with men is not explained by differences in comorbidity or differences in the effect of comorbidity on mortality.

The strength of the present study is the prospective inclusion of a large national cohort of patients starting LTOT for COPD with complete follow-up. The National Oxygen Register covers some 85% of all patients who have started LTOT since 1987 in Sweden, with loss of data from whole counties mainly due to temporary shortages of staff. [32]

Several potential weaknesses in the present study need to be mentioned. This study included patients starting LTOT and the distribution of comorbidities and their effects on mortality may not be representative for the whole population of patients with very severe COPD. Although diagnoses in the Swedish Hospital Discharge Register have been recently validated as having high specificity throughout as well as good sensitivity to certain conditions, such as cardiovascular disease, the register's sensitivity is lower for less acute or less serious conditions, such as hypertension and diabetes mellitus. [33] The sensitivity for cardiovascular disease might be lower in these patients with severe COPD than indicated by the validation studies, as cardiac disease may remain undiagnosed. [34] The misclassification from low sensitivity would likely tend to underestimate the association between these comorbidities and mortality, as unregistered comorbidity in the “healthy” group will make their mortality more similar to that of patients with registered comorbidity. [35] On the other hand, the estimates for comorbidities may be biased towards a higher effect, as patients with more severe illness may be more likely to be hospitalized and to get comorbidities registered. However, a too high effect of comorbidity is not a threat to the validity of the present finding that comorbidity does not explain the sex-related difference in mortality. We lacked data on prognostic predictors such as the level of dyspnea and exercise capacity, but inclusion of these factors, which are likely directly related to comorbidity, would have biased the associations between comorbidity and mortality. There were also insufficient data on duration and severity of comorbidities, but the sensitivity of the Discharge Register has been shown to be high for serious and more severe forms of comorbidity, [33] known to have prognostic impact in COPD. Thus, the present finding that comorbidity cannot explain sex-related differences in mortality is likely to have a high validity. In the authors' opinion, the mentioned potential weaknesses do not substantially alter the main finding of the present study.

What could be the mechanisms behind the survival difference between men and women in oxygen-dependent COPD, [4], [5], [6], [7] since it does not seem to be explained by comorbidity? The present data do not suggest that women started LTOT earlier in the course of their disease, or had less severe respiratory insufficiency, since FEV_1_% of predicted were similar and women in fact had a lower mean Pa_O2_ and a higher mean Pa_CO2_ on ambient air than men when starting LTOT. Whether the effect of LTOT or the compliance to the therapy differs between the sexes has not been evaluated because few women were included in the randomized trials and compliance has generally not been assessed. [2], [3] One hypothesis is that women, as compared to men, might develop COPD at lower smoking exposure [36] and report more dyspnea and lower quality of life independent of COPD severity. [27], [37] It is possible that women, due to less total smoking and different exposure to other biological factors (such as sex hormones) as well as environmental factors (such as alcohol, social and nutritional factors), might have lower frailty with the same degree of respiratory insufficiency than men after starting LTOT. There may also be phenotypical differences between men and women in COPD. Men have been found to have more emphysema than women at all levels of COPD severity and smoking, [38] which could contribute to the survival difference since the extent of emphysema predicts mortality. [39] Clearly, the sex-related survival difference in COPD with LTOT warrants further research.

For the clinician, this study shows that comorbidity has a major impact on mortality even in the most severe stages of COPD and highlights the importance of improved preventive treatments for conditions such as osteoporosis, as well as of optimized diagnosis and treatment of manifest comorbidity in order to improve the high morbidity and mortality in severe COPD.

In conclusion, this study shows that comorbidity differs between men and women, but differences in prevalence or effect of comorbidity do not explain the sex-related difference in mortality in oxygen-dependent COPD.

## Supporting Information

Table S1 Definitions of diagnosis entities and surgical procedures according to ICD9 (used before 1997) and ICD-10.
(DOC)

Table S2 Cox regression of overall mortality in patients (n = 7,593) who started LTOT 1995–2008. The analysis includes FEV_1_ in percent of predicted instead of absolute values, as well as BMI, which was available for this time period only.
(DOC)
