# Supplementary material for: Comorbidity and Sex-Related Differences in Mortality in Oxygen-Dependent Chronic Obstructive Pulmonary Disease
Source: PLoS One. 2012 Apr 26;7(4):e35806. doi: 10.1371/journal.pone.0035806 (PMC3338527; doi:10.1371/journal.pone.0035806)
Supplement: Table S1 — Definitions of diagnosis entities and surgical procedures according to ICD9 (used before 1997) and ICD-10. (DOC) [file pone.0035806.s001.doc]

**Table S1. Definitions of Diagnosis E**ntities

| **Diagnosis entity** | **ICD9** | **ICD-10** |
| --- | --- | --- |
| All cancers | 140-239 | C00-D48 |
| Anemia | 280-285 | D50-D64 |
| Arrhythmia | 426, 427 (excluding 427.5) | I44-I45, I47-I49 |
| Cerebrovascular disease | 430-438 | I60-I69 |
| COPD | 490-492, 495-496 | J40-J44 |
| Diabetes mellitus | 250 | E10-E14 |
| Digestive organ disease | 520-579 | K00-K93 |
| Heart failure | 428 | I50 |
| Hypertension | 401-405 | I10-I15 |
| Ischemic heart disease | 410-414 | I20-I25 |
| Lung cancer | 162 | C33-C34 |
| Lung transplantation | 335, 336 ^*^ | GDG ^*^ |
| Lung volume reduction surgery | 322 ^*^ | GDB ^*^ |
| Mental disorders | 290-319 | F00-F99 |
| Nonrespiratory disease | Not respiratory disease | Not respiratory disease |
| Osteoporosis | 733, V178.1, V828.1 | M80-M82 |
| Pulmonary embolism | 415.1 | I26 |
| Renal failure | 585-586 | N17-N19 |
| Respiratory disease | 277, 276C, 279, 786, 460-519 | E84, E87.2, E88.0, J00-J99, R04-R06 |
| Rheumatoid arthritis | 714, 720.0 | M05-M06 |

^*^ Surgical procedure codes.
