# Supplementary material for: Comorbidity and Sex-Related Differences in Mortality in Oxygen-Dependent Chronic Obstructive Pulmonary Disease
Source: PLoS One. 2012 Apr 26;7(4):e35806. doi: 10.1371/journal.pone.0035806 (PMC3338527; doi:10.1371/journal.pone.0035806)
Supplement: Table S2 — Cox regression of overall mortality in patients (n = 7,593) who started LTOT 1995–2008. The analysis includes FEV1 in percent of predicted instead of absolute values, as well as BMI, which was available for this time period only. (DOC) [file pone.0035806.s002.doc]

**Table S2. Cox regression, including FEV_1_% of predicted and BMI, of overall mortality in the subgroup of patients starting LTOT 1995 – 2008**

| **Characteristic** | **Hazard ratio** | **95% confidence interval** | **P-value** |
| --- | --- | --- | --- |
| Age | 1.04 | 1.04 – 1.05 | <0.001 |
| Female | 0.74 | 0.69 – 0.79 | <0.001 |
| Pa_O2_ air | 0.88 | 0.85 – 0.91 | <0.001 |
| FEV_1_, % of predicted | 1.00 | 0.99 – 1.00 | <0.001 |
| BMI ^*^ |  |  |  |
| 18.5 – 24.9 | 0.69 | 0.64 – 0.76 | <0.001 |
| 24 – 29.9 | 0.56 | 0.51 – 0.62 | <0.001 |
| ≥ 30 | 0.48 | 0.43 – 0.56 | <0.001 |
| Past smoking ^*^ | 1.02 | 0.88 – 1.18 | 0.806 |
| Current smoking ^*^ | 1.29 | 0.99 – 1.67 | 0.060 |
| Start year | 1.01 | 1.00 – 1.02 | 0.189 |
| Charlson score ^*^ |  |  |  |
| 1 | 1.16 | 1.07 – 1.25 | <0.001 |
| 2 | 1.48 | 1.33 – 1.65 | <0.001 |
| 3 | 1.66 | 1.41 – 1.94 | <0.001 |
| >3 | 1.89 | 1.56 – 2.29 | <0.001 |
| Anemia | 1.21 | 1.03 – 1.42 | 0.024 |
| Arrhythmia | 1.25 | 1.14 – 1.38 | <0.001 |
| Mental disorder | 1.09 | 0.96 – 1.23 | 0.193 |
| Osteoporosis | 1.29 | 1.13 – 1.47 | <0.001 |

Analysis of patients (n = 7,593) starting LTOT 1995 – 2008, as BMI was available during this time period only.

*Definition of abbreviations:* BMI = body mass index; FEV_1_ = forced expiratory volume in one second; Pa_O2_ air = arterial blood gas tension of oxygen while breathing ambient air.

^*^ The categories BMI < 18.5, never smoking, and Charlson score 0 were used as reference categories.
